# Effectiveness of Emotion‐Focused Therapy for Self‐Criticism: A Systematic Review and Meta‐Analysis

**DOI:** 10.1002/cpp.70346

**Published:** 2026-10-04

**Authors:** Yi Wang, Conor Douris, Ladislav Timulak

**Affiliations:** ^1^ School of Psychology Trinity College Dublin Dublin Ireland

**Keywords:** emotion‐focused therapy, meta‐analysis, self‐criticism

## Abstract

Problematic self‐criticism has been widely recognised as a transdiagnostic factor associated with a range of mental health disorders. Emotion‐focused therapy (EFT) targets self‐criticism primarily via two‐chair dialogue for self‐criticism, a core intervention within the approach. An increasing number of pre‐post studies and randomised controlled trials (RCTs) have examined the effectiveness of EFT in reducing self‐criticism. The present meta‐analysis synthesised existing quantitative studies utilising EFT to reduce self‐criticism and evaluated its overall effectiveness across study designs. Four pre‐post studies and seven RCTs were identified through a systematic literature search. Effect sizes were calculated separately for 11 within‐group pre‐post EFT intervention groups (four single‐arm pre‐post studies and seven RCT intervention groups) and seven between‐group RCT comparisons. Study quality, risk of bias and sensitivity analyses were conducted. Using random‐effects models, the pooled within‐group effect size (Hedges' *g*) was −0.86, and the pooled between‐group effect size in RCTs was −0.73, indicating moderate to large effects of EFT in reducing self‐criticism. Leave‐one‐out sensitivity analyses showed that these effects remained robust after the removal of outliers. This meta‐analysis provides the first quantitative synthesis of EFT for self‐criticism and offers preliminary evidence supporting its effectiveness in addressing this transdiagnostic factor linked to the number of mental health difficulties.

## Summary

- Emotion‐focused therapy (EFT) is associated with reductions in self‐criticism, with consistent effects observed across both randomised and pre‐post study designs.
- The findings suggest that EFT may facilitate change in maladaptive self‐relational processes linked to psychological distress.
- EFT shows potential as an effective intervention for reducing self‐criticism as a transdiagnostic factor, although further research is needed to strengthen the evidence base.

Emotion‐focused therapy (EFT; Greenberg and Korman 1993) is a research‐informed therapeutic approach that has been studied in its individual form, particularly in the context of depression, anxiety and complex trauma (e.g., Goldman et al. 2006; Paivio and Nieuwenhuis 2001; Timulak et al. 2022; Watson et al. 2003). This approach is now also being applied to other presentations such as eating disorders (Glisenti et al. 2021). EFT has been conceptualised as a transdiagnostic treatment model that addresses common problematic processes (Timulak and Keogh 2022). Within the EFT transdiagnostic conceptualisation, self‐criticism and its resulting maladaptive shame are seen as one of the theoretically central processes, occurring across a variety of difficulties.

Self‐criticism refers to a negative internal self‐evaluative system in which individuals tend to harshly judge themselves and hold a self‐deprecating manner (Gilbert et al. 2004; Shahar 2015). Rather than being confined to a single diagnostic category, increasing evidence has supported the view that self‐criticism is one of the transdiagnostic etiological factors contributing to the development and maintenance of various mental health disorders (McIntyre et al. 2018), including depression (Jhangir and Jameel 2023), social anxiety disorder (Iancu et al. 2015) and eating disorders (Paranjothy and Wade 2024). Importantly, self‐criticism is not only associated with symptom severity but also with psychotherapy processes and outcomes. For example, one meta‐analysis showed that higher levels of pretreatment self‐criticism are negatively correlated with treatment outcomes (Loew et al. 2020), highlighting its clinical relevance as a potential treatment target.

Individuals with high levels of self‐criticism tend to report greater self‐disgust and contempt, more sadness, increased submissiveness and heightened experiences of shame (Whelton and Greenberg 2005). Shame is widely regarded as an extremely painful and distressing self‐conscious emotion. A series of empirical studies have demonstrated that both external and internal shame are strongly correlated with self‐criticism (Gois et al. 2018; Oliveira et al. 2017). A recent network analysis examining the relationships among shame, self‐criticism, self‐compassion and self‐forgiveness found that comparative self‐criticism and internal self‐criticism were critical factors in the regulation and generation of shame, with comparative self‐criticism playing a particularly prominent role (ShamsAlam et al. 2025). Castilho et al. (2016) demonstrated that self‐criticism mediates the relationship between shame and psychopathology. Conversely, shame also mediates the association between self‐criticism and psychopathology. These findings suggest that shame and self‐criticism have a reciprocal relationship, reinforcing one another in a vicious cycle that perpetuates psychopathology.

EFT offers a distinct perspective for understanding emotional disorders that differs from more symptom‐focused psychotherapeutic approaches, including traditional cognitive behavioural therapy and some third‐wave approaches. Specifically, EFT conceptualises emotional difficulties by distinguishing between maladaptive primary and secondary emotions (Greenberg et al. 1996). Within the EFT framework, secondary emotions, such as global distress, rejecting anger and anticipatory anxiety, are understood as reactive and defensive responses to underlying primary emotional experiences that have not been adequately recognised or processed (Pascual‐Leone and Paivio 2013; Timulak and Pascual‐Leone 2015; Herrmann et al. 2016). Accordingly, EFT aims to facilitate access to and processing of underlying maladaptive primary emotions, including the maladaptive shame associated with self‐criticism. Therapeutic change occurs through the transformation of these maladaptive primary emotions by activating more adaptive emotional responses (Greenberg 2017; Timulak and Keogh 2022).

From an EFT perspective, self‐criticism is conceptualised as an internal conflict between two parts: the internal critical self and the experiencing self (Greenberg et al. 1996). When problematic self‐criticism is identified as an in‐session therapeutic marker, the two‐chair dialogue is employed as a core therapeutic task to help clients engage with and transform this internal self‐critical process (Elliott et al. 2025). During the two‐chair dialogue, clients alternate between the critical self and the experiencing self by moving from one chair to the other under the therapist's guidance. This process enables clients to explore, express and process the thoughts, emotions and unmet needs associated with each part (Elliott et al. 2004). Clients are encouraged to experience the impact of the critical voice, typically characterised by maladaptive shame, and to articulate the unmet needs of the experiencing self, such as needs for acceptance, understanding and support. The dialogue subsequently facilitates either the development of self‐compassion towards the criticised self or protective self‐assertion in response to the critic. Through the activation of these adaptive emotional responses, particularly protective anger and self‐compassion, maladaptive shame‐based emotional experiences are transformed, thereby facilitating the transformation of internal self‐criticism (Greenberg and Watson 2006; Timulak and Keogh 2020; Elliott et al. 2025). The task is based on a series of studies that started in the context of Gestalt therapy (e.g., Greenberg 1979, 1980, 1983).

Apart from EFT, several other therapeutic approaches have been developed to address problematic self‐criticism. For example, compassion‐focused therapy (CFT), informed by evolutionary psychology, attachment theory, Buddhist philosophy and social mentality theory (Gilbert 2014), and cognitive‐behavioural approaches targeting related constructs such as perfectionism (Galloway et al. 2022). Although both EFT and CFT recognise self‐compassion as an important therapeutic process in addressing problematic self‐criticism, their theoretical frameworks and mechanisms of change differ. Whereas CFT primarily addresses self‐criticism by developing a compassionate mind through techniques such as imagery, meditation, letter writing and role play (Gilbert 2009, 2014), EFT conceptualises self‐criticism as an internal conflict and facilitates emotional transformation by accessing and transforming the maladaptive primary emotions underlying self‐critical experience. Within this process, clients are guided to access and articulate their underlying unmet core emotional needs, thereby facilitating the emergence of adaptive emotional responses, particularly protective anger and self‐compassion (Greenberg and Watson 2006; Timulak and Keogh 2022).

Several meta‐analyses (e.g., Wakelin et al. 2022; Vidal and Soldevilla 2023; Millard et al. 2023) examined the effectiveness of CFT for self‐criticism, suggesting it is an effective approach for this type of clinical difficulty. Despite the fact that problematic self‐criticism constitutes a central focus within EFT theory and clinical practice, there have been no systematic reviews to date examining the effectiveness of EFT in addressing self‐criticism specifically. This represents an important gap in the existing literature. Given that EFT is conceptualised as a process‐based and transdiagnostic treatment model and that problematic self‐criticism together with the associated maladaptive shame are considered transdiagnostic common factors across a wide range of mental health difficulties, an investigation into the effectiveness of EFT for self‐criticism is warranted. A meta‐analysis can provide an important evidence base for clinical decision‐making regarding the use of EFT for this transdiagnostic therapeutic target. Based on the theoretical rationale and empirical evidence reviewed above, we hypothesised that EFT and EFT‐based interventions would be effective in reducing problematic self‐criticism. The present study therefore aimed to synthesise existing quantitative research on EFT and EFT‐based interventions specifically targeting self‐criticism, to evaluate their overall effectiveness and quantify the magnitude and examine the consistency of treatment effects across studies.

## 1 Method

The present systematic review and meta‐analysis were conducted and reported in accordance with the PRISMA 2020 statement (Page et al. 2021).

### 1.1 Literature Search

Literature searches were conducted through PsycINFO, PubMed, Web of Science and Scopus. To ensure a thorough and comprehensive retrieval of relevant studies, literature searches were carried out by the first and second authors. The first author conducted searches on 15 December 2024, 4 February 2025 and 15 April 2025, whereas the second author conducted a search on 17 April 2025.

An initial exploratory search was conducted using a range of phrases, including ‘emotion‐focused treatment and self‐criticism/self‐blame/inner critic’, ‘emotion‐focused intervention and self‐criticism/self‐blame/inner critic’ and ‘emotion‐focused approach and self‐criticism/self‐blame/inner critic’. These exploratory searches were conducted to assess the sensitivity and breadth of different search formulations rather than to define the final search strategy. Given that the present study specifically investigates the treatment effectiveness of EFT in reducing self‐criticism, several iterations were conducted. Ultimately, the terms ‘emotion‐focused’ and ‘self‐criticism’ were identified as the most efficient and specific and were therefore adopted as the final search terms, combined using the Boolean operator AND, to conduct a systematic search across the four main databases. The terms ‘self‐blame’ and ‘inner critic’, as well as descriptors such as ‘treatment’, ‘intervention’ and ‘approach’, were explored during the preliminary phase but were not used as search terms in the final database search. No restrictions were applied to the year of publication, provided that the terms ‘emotion‐focused’ and ‘self‐criticism’ appeared in the title or the abstract. Google Scholar, ResearchGate and the reference lists of relevant papers were also searched in addition to a more formal search within the four listed databases.

### 1.2 Eligibility Criteria

Eligibility criteria for studies in this meta‐analysis were based on quantitative research design, including randomised controlled trials and single‐group pre‐post studies. Specifically, studies were included if they (1) explicitly utilised EFT or an intervention based on EFT aimed at reducing self‐criticism (with self‐criticism as an explicit target or one of the explicit targets of the intervention) and (2) were quantitative and reported measurable changes in outcomes from pre‐ to post‐ intervention, including RCT studies and single group pre‐post studies. The studies should include specific data (i.e., means and standard deviations) necessary to calculate effect sizes; (3) utilised valid and reliable psychological assessment tools for measuring self‐criticism; and (4) were published in English in peer‐reviewed journals.

### 1.3 Selection Process

The selection of literature from key databases was conducted primarily with the aid of the online systematic review tool Rayyan (Ouzzani et al. 2016; see Figure 1) and combined with manual searches. After duplicates were systematically removed, 321 papers were excluded, leaving 520 studies for further screening. These studies were then examined to determine whether they employed quantitative intervention designs, resulting in the removal of 464 papers. The full texts of the remaining 56 papers were reviewed, and 44 were excluded because they either did not utilise an EFT intervention or the EFT intervention did not target self‐criticism. Among the remaining 16 papers, four were excluded because the papers did not report means and standard deviation, and when authors were contacted, they did not provide the requested information, two studies did not meet the predefined eligibility criteria and one study (Haberman et al. 2019) was excluded because its participants overlapped with those of an already included study (Shahar et al. 2017). Furthermore, Google Scholar, ResearchGate and the reference lists of relevant papers were also searched manually and a further two papers were included, leaving a total of 11 papers.

**FIGURE 1:**
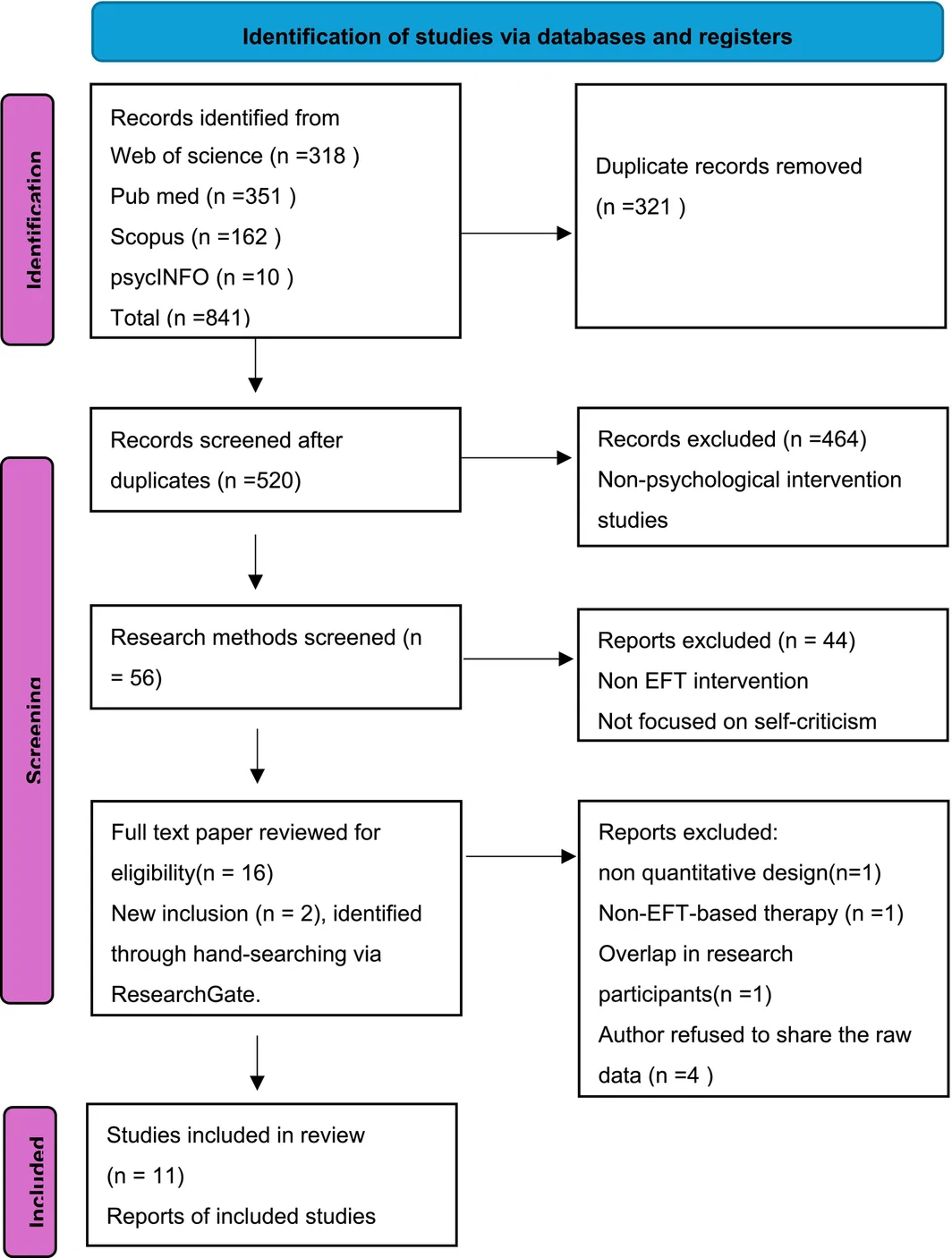
PRISMA flow diagram.

The second author independently replicated the search using the same key terms ‘emotion‐focused’ and ‘self‐criticism’, combined using the Boolean operator AND, across the four databases but followed a different screening sequence. This procedure identified 155 records by title, with 71 remaining after removing duplicates, 20 remaining after screening abstracts and 15 remaining after full‐text reviews. Following a second, more detailed full‐text appraisal, five additional studies were excluded, leaving 10 studies matching exactly the final set obtained in the primary search. The final selection of the studies was also discussed with the third author, and decisions regarding inclusion or exclusion were made based on the predefined eligibility criteria. Studies that met the inclusion criteria but lacked critical data and failed to provide it after contacting the original authors via email were excluded from the final analysis.

### 1.4 Data Extraction

The following information was extracted for each included study (see Table 1): (a) research design (randomised controlled trial or single‐group pre‐post study); (b) assessment time points (pre‐intervention, post‐treatment and any follow‐up assessments); (c) participant‐recruitment methods; (d) participant characteristics, specifying whether the sample was clinical or nonclinical and detailing presenting problems; (e) sample size and gender distribution; (f) intervention format, content and duration (i.e., core EFT components delivered in group therapy, individual therapy or group‐training formats); (g) overall length of the EFT intervention; (h) self‐criticism measures employed; (i) country of origin and participants' ethnicity; and (j) means and standard deviations required to calculate effect sizes, primarily the pre‐ and post‐ intervention means and standard deviations and (k) treatment completion and dropout rates.

**TABLE 1:** Summary of studies on emotion‐focused interventions for self‐criticism.

Study	Research design	Time points	Recruitment method	Participants	Sample (*N*)	Gender (%)	Intervention format	Duration	Measure	Country
Shahar et al. (2011)	Single group (pre‐post)	Baseline, post, 2/4/6‐mo FU	Community advertisement	Highly self‐critical adults (clinical interview, FSCRS ≥ 1 SD)	10	100% women	Individual EFT therapy	5–8 sessions	FSCRS‐IS	Israel/USA
Thompson and Girz (2019)	Single group (pre‐post)	Baseline, post‐R1/2 (4‐wk)	University counselling referral	Students with depression/anxiety and self‐criticism	Round 1: 37; Round 2: 22	79% women; 21% men	EFT Group therapy	Weekly sessions	BDI	Canada
Stiegler et al. (2017)	Single‐group (pre‐post)	Pre, mid, post	Public programme	Clients with depression/anxiety (on sick leave, BDI/BAI)	21	71.4% women, 28.6% men	Individual EFT therapy	5–9 baseline +5 sessions	FSCRS	Norway
Shahar et al. (2017)	Single‐group (pre‐post)	Baseline, post, 6/12‐mo FU	Announcements and listservs	Adults with SAD (MINI, SPIN > 28)	12	41.7% women, 58.3% men	Individual EFT therapy	28 sessions	FSCRS‐IS	Israel
Baránková and Sládkovičová (2022)	RCT (exp vs. control)	Pre, post	Social networks	Women with body image dissatisfaction (BSQ ≥ 52)	64 (32 per group)	100% women	EFT‐EC	14 daily practices	FSCRS‐IS	Slovakia
Halamová et al. (2019)	RCT (3 arms)	Pre, post	University students	Psychology students (course credit)	73 (19 int., 20 act ctrl, 34 no treat)	89% women, 11% men (intervention)	EFT‐EC	12 weekly + daily	FSCRS‐IS	Slovakia
Baránková and Vaďurová (2022)	RCT (exp vs. control)	Pre, post	General population	Adults with high narcissism (NPI > average)	63 (32 int., 31 ctrl)	68.8% women, 31.2% men	EFT‐EC	14 daily practices	FSCRS‐IS + HS	Slovakia
Baránková and Karpinský (2022)	RCT (exp vs. control)	Pre, post	Social networks	Adults with mild internet addiction (IAT ≥ 31)	67 (37 int., 30 ctrl)	Int: 75.7% w/Ctrl: 56.7% w	EFT‐EC	14 daily practices	FSCRS‐IS + HS	Slovakia
López Cavada et al. (2024)	RCT (int vs. waitlist)	Baseline, post, 3‐mo FU	Social media ad	Nonclinical adults with high self‐criticism (FSCRS ≥ 1 SD)	52 (24 int., 28 ctrl)	Int: 62.5% w/Ctrl: 82.1% w	Individual EFT for self‐criticism	10–12 sessions	FSCRS‐IS	Spain
Salasi et al. (2023)	RCT (EFT vs. SCT vs. control)	Pre, post, 3‐mo FU	Addiction clinic	Methadone‐maintained patients	45 (15 per group)	53.3%–60% men across groups	Individual EFT vs. SCT	12 sessions	FSCRS	Iran
Halamová et al. (2022)	RCT (exp vs. control)	Pre, post	Social networking	Helping professionals	65 (32 int., 33 ctrl)	97% women	EFT‐EC	14 daily practices	FSCRS‐IS	Slovakia

Abbreviations: act ctrl, active control; BAI, Beck Anxiety Inventory; BDI, Beck Depression Inventory; BL, baseline; BSQ, Body Shape Questionnaire; ctrl, control; EFT, emotion‐focused therapy; EFT‐EC, emotion‐focused training for self‐compassion and self‐protection; exp., experimental; FSCRS, Forms of Self‐Criticising/Attacking and Self‐Reassuring Scale; FSCRS‐IS, FSCRS Inadequate Self Scale; FSCRS‐HS, FSCRS Hated Self Scale; FU, follow‐up; IAT, internet addiction test; IEFT, intensive emotion‐focused therapy; int., intervention; M, men; MINI, Mini‐International Neuropsychiatric Interview; mo, month; no treat, no treatment; NPI, Narcissistic Personality Inventory; post, posttreatment; R, round; RCT, randomised controlled trial; SAD, social anxiety disorder; SCT, schema therapy; SPIN, Social Phobia Inventory; W, women; wk., week; WL, waitlist.

Of the 11 included studies, 10 employed the Forms of Self‐Criticism and Self‐Reassurance Scale (FSCRS; Gilbert et al. 2004), a self‐report questionnaire designed to assess levels of self‐criticism. However, researchers differed in their preferred scoring approach. Some adopted the overall FSCRS score to measure self‐criticism. In contrast, others were more inclined to use specific subscales, most commonly the inadequate self (IS) subscale, or in some cases, a combined score of IS and hated self (HS). The HS subscale is typically associated with more severe clinical presentations and shows a floor effect in nonclinical populations (Baião et al. 2014). Given that the majority of participants in the included studies were not clinical patients with formally diagnosed mental health disorders or rigorous clinical assessment, the IS subscale was extracted and used to assess self‐criticism whenever specific values were available.

For three studies, IS subscale data were not available. In these three cases, we did the following: For Thompson and Girz (2019), no self‐criticism measure such as the FSCRS was reported. Therefore, the total BDI (Beck Depression Inventory) score, which was the primary outcome reported in the study, was extracted for the analysis (Beck et al. 1996). Stiegler et al. (2017) explicitly stated they used the IS subscale to measure self‐criticism but did not report the mean and standard deviation of pre‐ and post‐intervention; the authors later provided the overall FSCRS scores by email upon request. Furthermore, Baránková and Vaďurová (2022) clarified that they used a combined IS + HS score (excluding items 12 and 22) to assess participants' self‐criticism (personal communication). In addition, Baránková and Sládkovičová (2022) did not report the pre‐treatment mean and standard deviation for the EFT intervention group. Because the pre‐intervention and pre‐control means did not differ significantly and the two groups contained the same number of participants, we used the control group's pre‐treatment mean and standard deviation as a proxy for the missing intervention group baseline values.

### 1.5 Risk of Bias Assessment of Included Studies

Because the review includes four single‐group pre‐post studies and seven RCT studies, risk of bias was evaluated differently due to different research designs. Different appraisal tools were applied in line with established methodological recommendations.

The risk of bias assessment for the seven RCT studies was conducted based on the principles of the Revised Cochrane Risk‐of‐Bias Tool for Randomised Trials (RoB 2; Sterne et al. 2019), which assesses five domains: (1) the randomisation process, (2) deviations from the intended interventions, (3) missing outcome data, (4) measurement of the outcome and (5) selection of the reported results.

Based on recommendations outlined in the review study by Ma et al. (2020) on research methodological quality assessment tools, the ‘Quality Assessment Tool for Before‐After (Pre‐Post) Studies With No Control Group’ was adopted and utilised to assess the quality of the single‐group pre‐post studies included in this meta‐analysis (National Heart, Lung, and Blood Institute [NHLBI], n.d.).

The overall evaluation process for the risk of bias assessment in the RCT studies and the quality assessment of the pre‐post studies was conducted independently by the first and second author. They agreed exactly in 9 out of 11 included studies. Disagreements regarding the two remaining studies were resolved through discussion, and consensus was achieved in all cases.

### 1.6 Effect Size Calculation

The standardised mean difference (SMD) Hedges' *g*, a corrected version of Cohen's *d* was used as the effect size as it reduces bias associated with small sample sizes (Durlak 2009). All relevant raw data, including pre‐ and post‐intervention means, pre‐ and post‐intervention standard deviations, and sample sizes were organised, synthesised and entered into R (R Core Team 2024). Data synthesis for the meta‐analysis was conducted using RStudio 2024 (see Table S1). Given that the SMD does not account for the correlation between pre‐ and post‐measures, which may bias the estimation of effect sizes, standardised mean change (SMC, for details see below) was used for the analysis of single‐arm pre‐post studies (Morris and DeShon 2002).

The calculation of effect sizes in this meta‐analysis was divided into two parts. For within‐group studies (pre‐ and post‐EFT intervention group), effect sizes were calculated by dividing the mean change (pre‐ to post‐intervention) by the pooled standard deviation. However, none of the included studies reported the correlation between pre‐ and post‐intervention scores for the EFT interventions. Morris and DeShon (2002) noted that the value of *r* varies depending on study characteristics, such as the timing of assessments, and can substantially influence effect size estimates. Therefore, effect sizes for within‐group studies were calculated using three assumed pre‐post correlation coefficients (*r* = 0.3, 0.5, and 0.7). For the RCT between‐group studies, effect sizes were calculated based on the mean difference between the postintervention experimental group and the postintervention control group, divided by the pooled standard deviation (SMD).

The overall pre‐post effect sizes were synthesised not only from single‐arm studies but also from RCT EFT intervention pre‐post groups to evaluate the overall effectiveness of EFT for self‐criticism. Due to the variation in clinical characteristics and research designs among the included studies, the calculation of overall effect sizes, whether from RCT studies or single‐arm pre‐post studies (including RCT EFT intervention groups), was based on a random‐effects model, given potential heterogeneity across studies (e.g., differences in participant characteristics, duration, study quality and format of interventions). The purpose of adopting this approach is to enhance the generalizability and persuasiveness of the findings (Higgins et al. 2020).

### 1.7 Publication Bias

Publication bias is a phenomenon that is difficult to eliminate in systematic reviews and meta‐analyses due to academic peer reviewers' preference for positive (clinically significant) findings, which can lead to difficulties in obtaining negative research findings from real clinical practice (Nair 2019). This meta‐analysis adopted a funnel plot to evaluate the publication bias through the observation of symmetry of the funnel plot. However, the use of visual observations to determine publication bias can be subjective. In an effort to increase objectivity, this meta‐analysis also evaluated publication bias using Egger's regression test (Egger et al. 1997), Begg's rank correlation test (Gjerdevik and Heuch 2014) and trim‐and‐fill analyses (Duval and Tweedie 2000) to better understand its impact on robustness of the estimated effect size.

### 1.8 Sensitivity Analysis

Given the small number of studies and thus a possible impact of heterogeneity of the effect sizes on the overall outcome, the leave‐one‐out method was adopted to examine each included study's effect on the overall estimated effect size. This was done by sequentially excluding each study one at a time. The aim was to rule out the potential outlier study's effect on the robustness of the findings, thereby increasing the transparency and credibility of this meta‐analysis and enhancing the reliability and generalisability of the findings (Mathur and VanderWeele 2020).

### 1.9 Moderator Analysis

In this meta‐analysis, after conducting the baseline random‐effects meta‐analysis, a moderator analysis was conducted separately for the RCT and pre‐post studies. The moderators included gender (percentage of women participants), duration of intervention (number of sessions), format of intervention (EFT training, individual intervention and group therapy), measurement (FSCRS, IS and IS + HS), sample size, quality of study and country. For these moderators, univariate mixed‐effects meta‐regression and subgroup analyses were conducted to explore the potential moderators' effect on heterogeneity and their potential influence on the estimated effect size.

## 2 Results

### 2.1 Description of Included Studies

The meta‐analysis included 11 studies that met the eligibility criteria, comprising seven randomised controlled trials and four single‐group pre‐post studies. The interventions included individual EFT, group EFT and EFT‐training interventions targeting self‐criticism. Participants comprised both clinical and nonclinical populations presenting with elevated self‐criticism or a range of psychological difficulties, including depression, anxiety, social anxiety disorder, body image dissatisfaction, narcissistic traits, internet addiction and methadone‐maintained patients. Intervention duration varied considerably across studies, ranging from 14‐day training programmes to 28‐session psychotherapy interventions.

All included studies were published before 17 April 2025 and were conducted across seven countries, including two from North America, three from Europe, one from Israel and one from Iran. A total of 531 participants were included, of whom 87% were women, with two studies recruiting exclusively female participants. Most studies recruited participants through community advertisements, social media or university settings. Nine of the 11 studies reported dropout rates below 20%, whereas one study (Halamová et al. 2022) reported a substantially higher dropout rate of 74.8%. Detailed characteristics of the included studies are presented in Table 1.

### 2.2 Risk of Bias Assessment

The final results of the risk of bias assessment for the RCT studies are presented in Table S2. Overall, five of the seven RCTs were judged as having a high risk of bias, one study was judged as having some concerns and one study was judged as having a low risk of bias. A review of the RCTs included in this meta‐analysis revealed several recurring methodological concerns. Many papers mentioned that participants were randomly assigned to intervention and control groups but generally failed to describe the randomisation procedure in detail. Specifically, Baránková and Sládkovičová (2022), Halamová et al. (2019), Baránková and Vaďurová (2022), Baránková and Karpinský (2022) and Halamová et al. (2022) did not clearly describe the randomisation procedure or allocation concealment. Several trials analysed only those participants who completed the intervention and posttreatment questionnaires, excluding dropouts and failing to report reasons for attrition. Baránková and Sládkovičová (2022) and Halamová et al. (2019) did not clearly report intention‐to‐treat analyses and provided limited information regarding the handling of missing outcome data. Furthermore, some studies did not apply appropriate statistical methods to handle missing participants and generally did not follow the intention‐to‐treat principle. Many studies did not register or report their planned research methods in advance. Five of the included RCTs originated from two research teams, three came from Baránková's research team and two from Halamová's research team. Their research methods and intervention contents (Emotion‐Focused Training, EFT‐SCP) were almost identical, with the main differences being the participants. Moreover, most of the studies assessed as high risk came from these two research teams. Among the seven RCTs, the studies by López‐Cavada et al. (2024) and Salasi et al. (2023) stand out for their stronger study design, clearer description of the intervention procedures and more comprehensive reporting of data‐analysis methods and results.

The final quality assessment of the four single‐arm pre‐post studies is presented in Table S3. Overall, one study was rated as good quality, and three studies were rated as fair quality. A widespread limitation in the four single‐arm pre‐post research designs was the relatively small sample sizes. None of the four single‐group pre‐post studies met the blinding criterion, which means that expectancy effects from the therapist and clients could have influenced the reliability of their outcomes. More detailed information can be found in Table S3.

### 2.3 Random‐Effects Model for Pre‐Post Effect Size on Self‐Criticism

The analysis was based on all 11 EFT intervention groups (four single‐arm pre‐post studies and seven RCT intervention groups). Given that the pre‐ and post‐intervention comparisons do not constitute a between‐group research design, we initially selected a correlation coefficient of *r* = 0.5 to estimate the SMD (Hedges' *g*). This choice was informed by the sensitivity analysis presented in Table S4, which tested the robustness of the effect size estimates across three assumed values of *r* (0.3, 0.5 and 0.7). When *r* = 0.3, the pooled effect size (Hedges' *g*) is −0.7320 (95% CI, −0.9822 to −0.4819; *p* = 0.0009), *I* ^2^ = 66.33%; when *r* = 0.5, the pooled effect size (Hedges' *g*) is −0.8638 (95% CI, −1.1551 to −0.5725; *p* = 0.0001), *I* ^2^ = 73.39%; when *r* = 0.7, the pooled effect size (Hedges' *g*) is −1.0999 (95% CI, −1.4615 to −0.7383; *p* < 0.0001), *I* ^2^ = 80.40%. These results demonstrated that the effect size varied depending on the assumed correlation coefficient. For each specific study, the variation in effect size under different assumed values of *r* is shown in Figure S1.

The value of Hedges' *g* (−0.7320 to −1.0999) increases with the increasing value of *r* (0.3 to 0.7). Further sensitivity analysis revealed that the trend of the findings and the significance of the *p* values remain unchanged with an increased value of *r*. However, the values of *I* ^2^ increased (66.33%–80.40%) with the increase in the value of *r*. Given the robustness of the findings, we decided to choose *r* = 0.5 as the most appropriate value, avoiding both overestimating and underestimating the effect size of the pre‐post EFT intervention for self‐criticism (Balk et al. 2012).

The results of the effect size synthesis of the single‐group pre‐post EFT intervention group based on the random effects model can be seen in Figure 2. As explained above, the rationale for choosing *r* = 0.5 as the assumed correlation coefficient to calculate the effect size of the pre‐post EFT intervention has been provided. Hence, the effect size of the single‐group pre‐post EFT for self‐criticism reached a large effect with Hedges' *g* = −0.86, *r* = 0.5, 95% CI −1.1551 to −0.5725.

**FIGURE 2:**
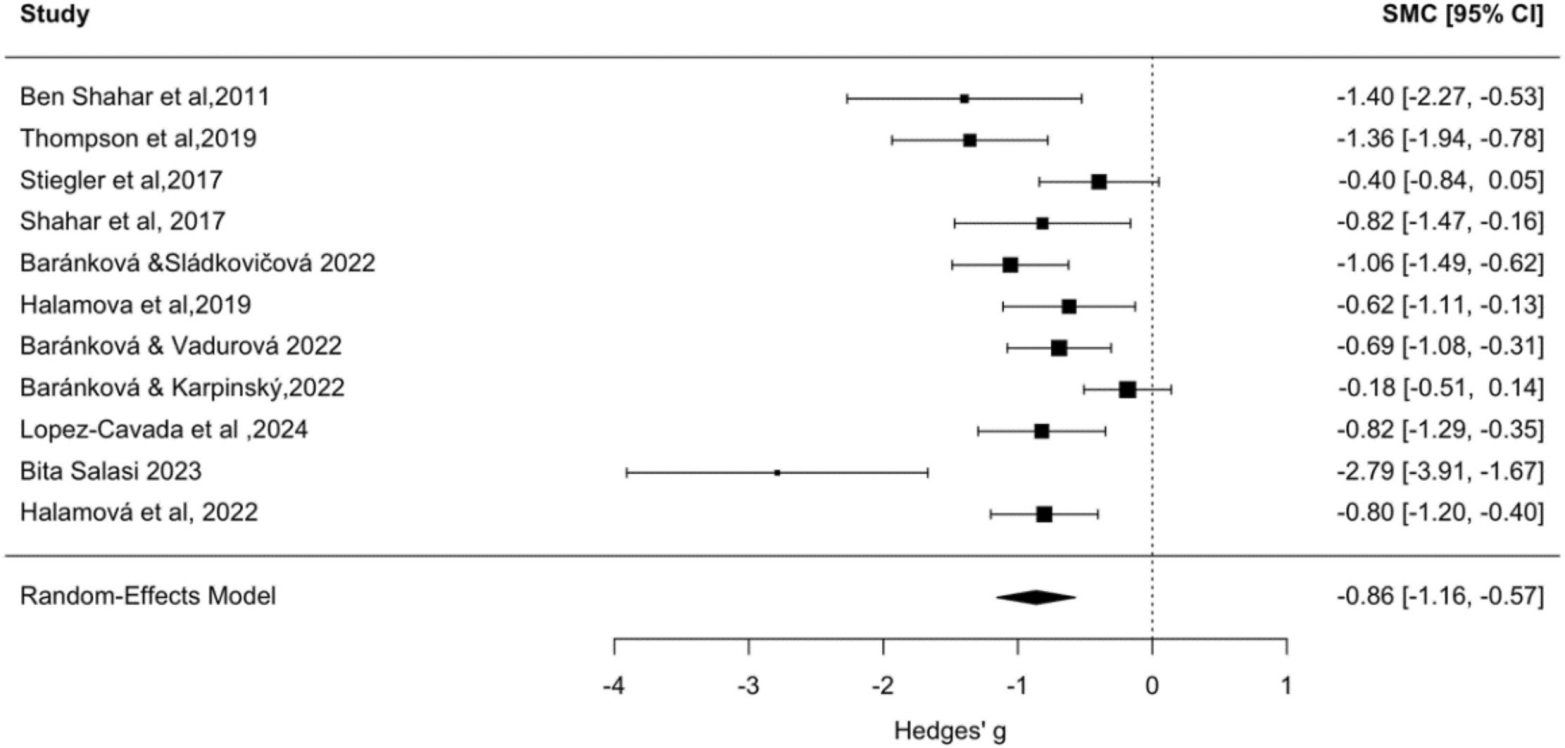
Pre‐post EFT intervention random‐effect model for self‐criticism.

### 2.4 Random‐Effects Model for Between‐Group Effect Size on Self‐Criticism

The present RCT meta‐analysis included seven studies. Raw data of the control groups (postmean, poststandard deviation and *N*) and intervention groups (postmean, poststandard deviation and *N*) were extracted to calculate the between‐group effect size based on the random effects model. Overall, the effect size of the meta‐analysis of seven RCT studies reached moderate to large with Hedges' *g* = −0.74, 95% CI: −0.99 to −0.48 (see Figure 3), which is statistically significant, and the confidence interval did not cross zero, which rejects the null hypothesis of no treatment effect.

**FIGURE 3:**
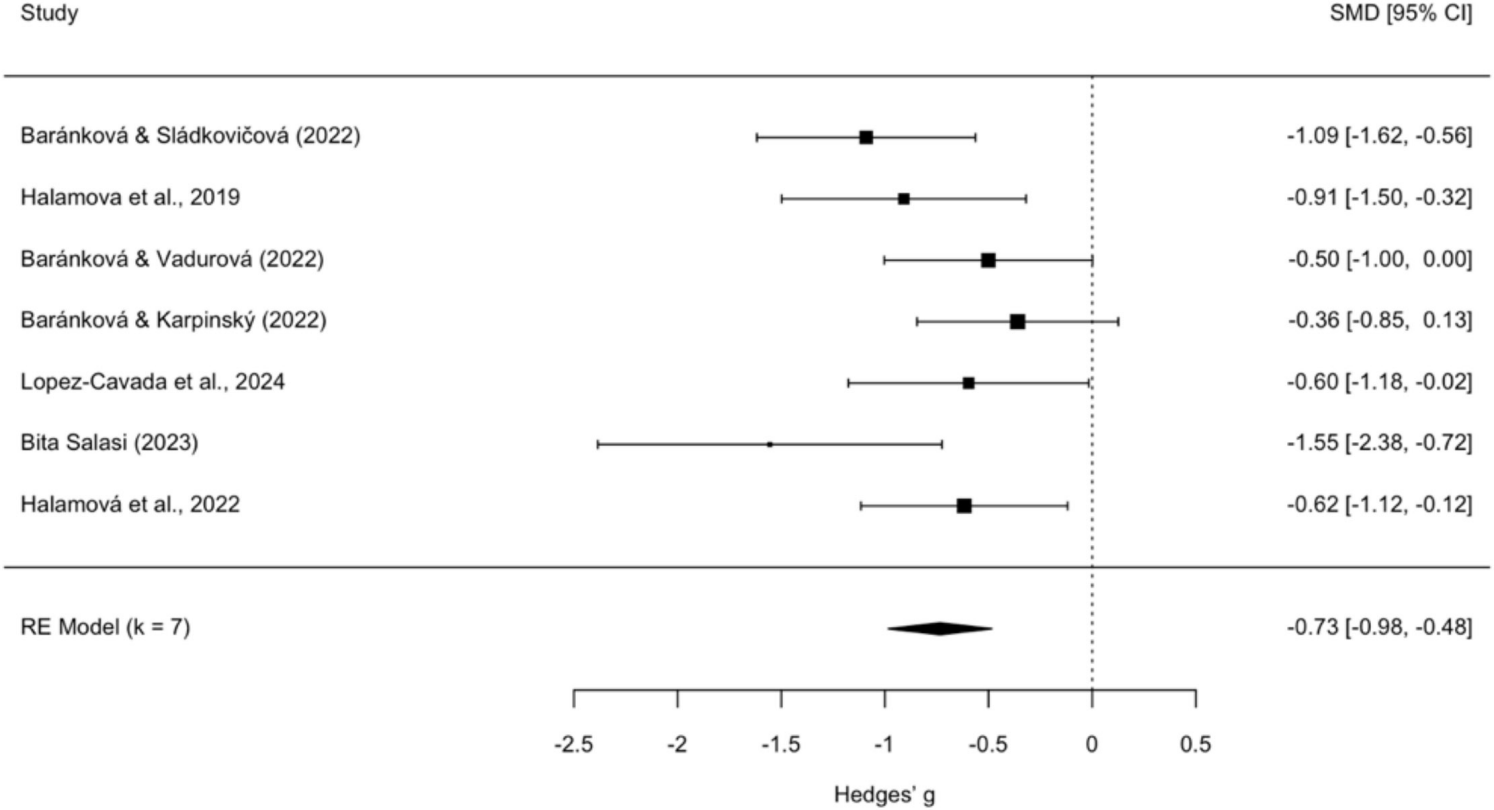
Random‐effects model of EFT intervention in RCT studies for self‐criticism.

### 2.5 Publication Bias

With regard to the single‐group pre–post studies, as shown in the funnel plot (see Figure 4), the distribution of studies appears to be asymmetrical regarding the overall effect size, suggesting potential publication bias. Egger's regression test (*z* = −4.1951, *p* < 0.0001) and Begg's rank correlation test (Kendall's *τ* = −0.4909, *p* = 0.0405) both indicate significant evidence of publication bias in the current single‐group pre‐post meta‐analysis (due to Salasi et al. 2023 study). After applying the trim‐and‐fill method to adjust the effect size, the result showed that no missing studies were imputed on the right side, and the adjusted effect size (Hedges' g = −0.8638, SE = 0.1486, 95% CI: −1.1551 to −0.5725, *p* < 0.001) remained consistent with the original findings. This suggests that the overall effect size was minimally affected by potential outliers.

**FIGURE 4:**
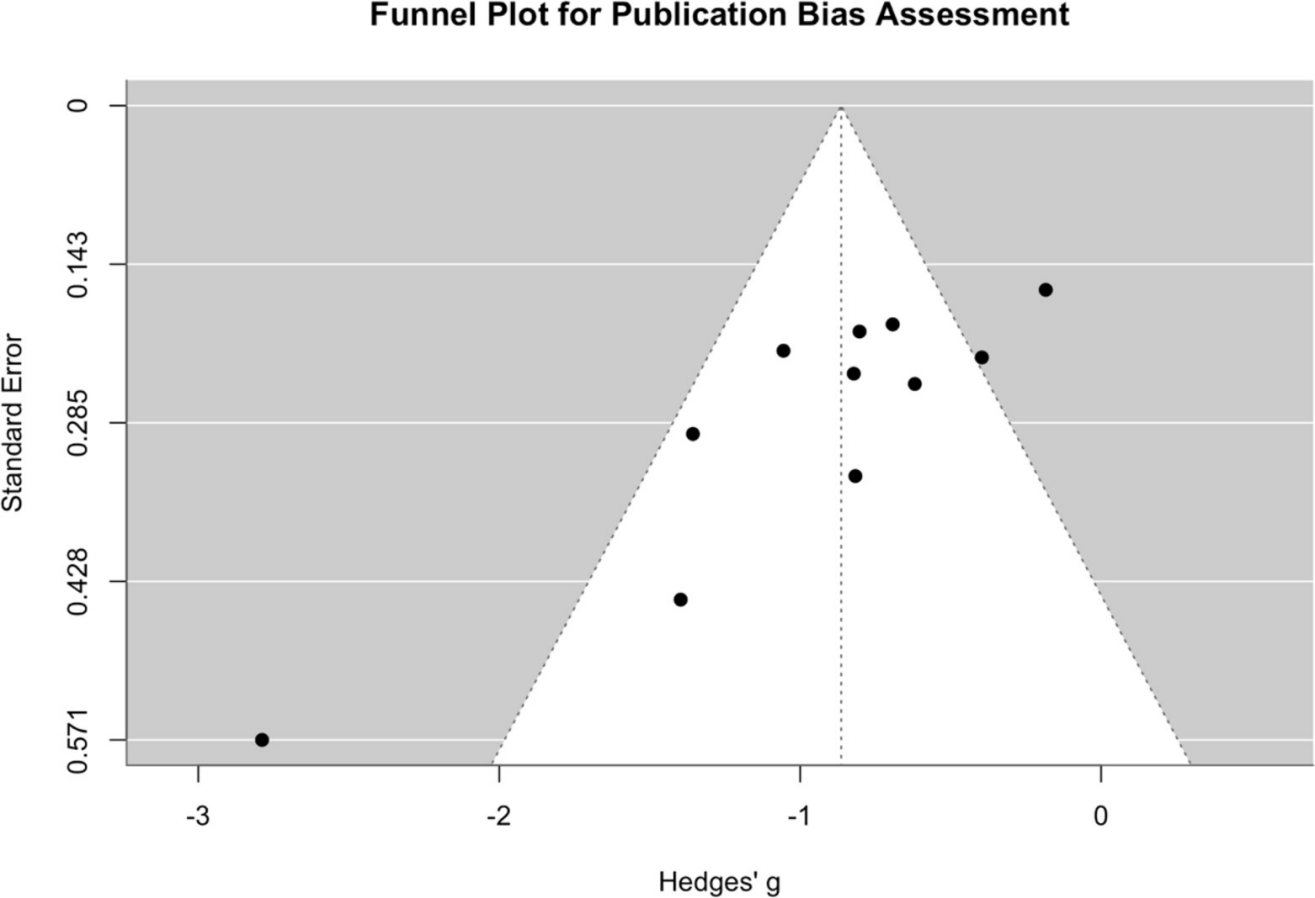
Funnel plot of pre‐post EFT intervention for self‐criticism.

In order to evaluate the publication bias of RCT studies, the funnel plot, Egger's regression analysis, Begg's correlation analysis and a trim‐and‐fill analysis were adopted. Firstly, as shown in Figure 5, the research is relatively asymmetrically distributed regarding the estimated overall effect size. The result of Egger's regression analysis is *z* = −2.3751, *p* = 0.0175, *p* < 0.05, indicating statistically significant asymmetry in the funnel plot. However, Begg's test did not find significant publication bias (Kendall's *τ* = −0.6190, *p* = 0.0690). Although Begg's test did not yield statistically significant results, the number of included studies was fewer than 25, and therefore, the possibility of publication bias cannot be ruled out (Begg and Mazumdar 1994). The trim‐and‐fill method was adopted to adjust for potential publication bias. The adjusted overall effect (Hedges' *g* = −0.7368, 95% CI: −0.9888 to −0.4848) was nearly identical to the original estimate, indicating that the estimated effect size was not influenced by potential publication bias.

**FIGURE 5:**
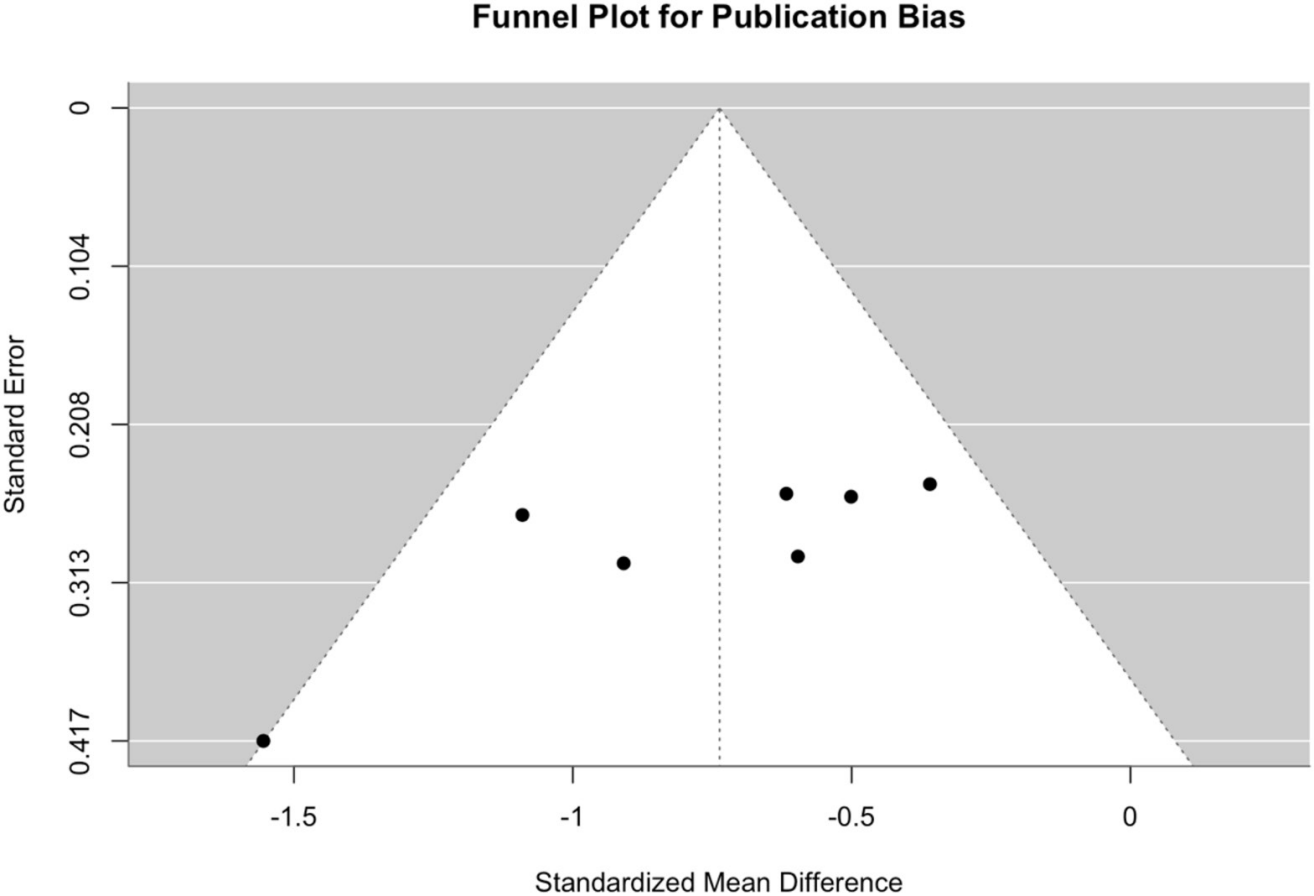
Funnel plot of RCT EFT intervention for self‐criticism.

### 2.6 Sensitivity Analysis

A sensitivity analysis was conducted in response to the results of Egger's regression tests, which showed that the results of single‐group pre‐post studies and RCT studies reached statistical significance. Sensitivity analysis of this meta‐analysis mainly focused on the outlier studies, especially those studies whose 95% CI crossed zero and whose 95% CI was relatively wide. These characteristics may indicate potential Zhong'Wen confounding variables that compromise the reliability of the results.

In relation to the single‐group pre‐post studies, three studies were removed from the meta‐analysis: the outlier study by Salasi et al. (2023) and two studies (Baránková and Karpinský 2022; Stiegler et al. 2017) whose 95% confidence intervals (CI) crossed zero and therefore indicated nonsignificant results. The remaining eight studies were synthesised to recalculate the effect size, showing that the effect size (Hedges' *g* = −0.87, *r* = 0.5, 95% CI: −1.05 to −0.70) is nearly identical to the original result after removing the outliers and nonsignificant studies.

When only the outlier study by Salasi et al. (2023) was removed, the pre‐‐post effect size decreased from Hedges' *g* = −0.86 (*r* = 0.5, 95% CI: −1.1551 to −0.5725, *I* ^2^ = 73.39%) to Hedges' *g* = −0.75 (*r* = 0.5, 95% CI: −0.98 to −0.52, *I* ^2^ = 57.9%). This indicates that the overall effect size was slightly impacted by Salasi et al. (2023), which had the largest effect size in this meta‐analysis. However, the general effect size remained moderate to large, and the result remained significant.

To further assess the robustness of the findings, the leave‐one‐out method was conducted to examine the influence of each included study on the overall estimated effect size, sequentially excluding each study one by one. As shown in Figure S2, the overall effect size was slightly influenced by Salasi et al.'s (2023) study compared to the removal of other included studies. Nevertheless, the revised pooled effect size remained in the moderate‐to‐large range and was still statistically significant.

In relation to the RCT studies, the study by Baránková and Karpinský (2022) was excluded due to its nonsignificant findings and Salasi et al. (2023) due to the relatively wide 95% confidence interval. The recalculated effect size, based on the remaining five studies and using a random‐effects model, was Hedges' *g* = −0.73 (95% CI: −0.97 to −0.49), which closely aligned with the original estimate. When only Salasi et al. (2023) was excluded, the effect size decreased slightly from Hedges' *g* = −0.73 (95% CI: −0.98 to −0.48, *I* ^2^ = 26.1%) to Hedges' *g* = −0.66 (95% CI: −0.88 to −0.44, *I* ^2^ = 3.2%). Similarly, excluding the outlier study by Baránková and Sládkovičová (2022) resulted in a marginally reduced effect size of Hedges' *g* = −0.65 (95% CI: −0.88 to −0.42, *I* ^2^ = 0.2%). These results suggest that although these outlier studies have only a modest impact on the pooled effect size, they contribute substantially to the observed heterogeneity (see Figure S3).

Overall, the sensitivity analyses demonstrated that, although heterogeneity was reduced after excluding the outlier studies, the pooled effect sizes changed only modestly and remained in the moderate‐to‐large range. As the overall conclusions remained robust, the original random‐effects models including all eligible studies were retained as the primary analyses.

### 2.7 Moderators Analysis

The baseline random‐effects meta‐analysis revealed that the pooled effect size for single‐group pre‐post studies was Hedges' *g* = −0.86, with substantial heterogeneity (*I* ^2^ = 73.39%). To explore potential sources of this heterogeneity, multiple univariate meta‐regression analyses were conducted using study‐level variables, including intervention format, intervention duration, sample size, gender distribution, measurement tool and study quality. None of these moderators significantly predicted effect size variation. The observed heterogeneity appears more likely to be attributable to statistical outliers than to differences in measurement tools or study design. Sensitivity analysis confirmed that heterogeneity was substantially reduced after removing outlier studies, further supporting this interpretation.

## 3 Discussion

### 3.1 Clinical Implications

Currently, most studies on transdiagnostic shared mechanisms have been conducted within the context of aetiology‐based transdiagnostic cognitive behavioural approaches, including selective attention, rumination, metacognitive beliefs and experiential avoidance (Harvey et al. 2004; Schaeuffele et al. 2024). From the perspective of experiential therapies such as EFT and CFT, self‐criticism is increasingly regarded as an important transdiagnostic treatment target. A growing body of research suggests that elevated self‐criticism contributes to the development and maintenance of a wide range of mental health disorders (McIntyre et al. 2018).

Research on EFT interventions targeting self‐criticism has gained increasing academic attention since 2010, with a notable rise in English‐language publications from 2020 onwards. To our knowledge, this is the first meta‐analysis to systematically evaluate the effectiveness of EFT in reducing self‐criticism. Given the limited number of RCTs, the meta‐analysis included both single‐group and RCT designs. The pooled effect size for pre‐post studies was Hedges' *g* = −0.86, and the pooled between‐group effect size from RCTs was Hedges' *g* = −0.73. Sensitivity analyses indicated that these effects remained in the moderate‐to‐large range after removing statistical outliers. These findings tentatively support the potential utility of EFT as an intervention for reducing self‐criticism.

### 3.2 Methodological Considerations and Limitations

A particular methodological feature of this study was the inclusion of both pre‐post and between‐group designs. For the within‐group meta‐analysis, we synthesised pre‐post data not only from four single‐group pre‐post studies but also from the EFT arms of seven RCTs to generate a more comprehensive estimate. However, as Cuijpers et al. (2016) noted, pre‐post effect sizes provide limited insight into the true efficacy of an intervention, as such designs do not control for confounding factors such as spontaneous remission, natural recovery or time effects. Furthermore, pre‐post studies are particularly vulnerable to expectancy effects, whereas RCTs are better positioned to distribute such biases across conditions. These concerns were evident in our findings, as substantial heterogeneity was observed in the effect size estimates from the pre‐post studies.

A further limitation of pre‐post designs lies in the frequent absence of reported pre‐post correlation coefficients, which necessitates estimation based on assumed values. Several previous meta‐analyses have adopted *r* values ranging from 0.7 to 0.75 (e.g., Hofmann et al. 2014; Johnsen and Friborg 2015). As a methodological contribution, the present study calculated effect sizes under three assumed correlation coefficients (*r* = 0.3, 0.5, 0.7). In line with Balk et al. (2012), who reported a median within‐group correlation of *r* = 0.59 across 811 studies, we adopted r = 0.5 as a conservative default. To assess the robustness of the results, we also conducted sensitivity analyses under all three *r* values for each included study.

This meta‐analysis revealed evidence of potential publication bias, as indicated by Egger's regression test. Publication bias is a well‐documented concern in psychological research, where studies reporting statistically significant findings are more likely to be published (Nair 2019). Such bias may result in the overestimation of treatment effects in meta‐analyses (Thornton 2000). These results should therefore be interpreted with appropriate caution, particularly given the possibility of inflated effect size estimates.

### 3.3 Comparison With Previous Studies

To date, meta‐analytic evidence specifically evaluating interventions for reducing self‐criticism remains limited, with existing meta‐analyses primarily focusing on compassion‐based interventions. In considering the clinical significance of these findings, it is informative to compare them with similar interventions such as CFT. For example, Wakelin et al. (2022) reported a moderate effect size of Hedges' *g* = 0.51 for self‐compassion‐related interventions in reducing self‐criticism across 20 RCTs. Vidal and Soldevilla (2023), focusing on the IS subscale, found a between‐group effect size of *d* = 0.30. Similarly, Millard et al. (2023) reported small effect sizes in clinical samples using the HS (*g* = −0.34) and IS (*g* = −0.28) subscales. Although these CFT studies appear to show smaller effects than those found in the present meta‐analysis, any cross‐study comparison must be approached with caution due to differences in study design, participant characteristics, intervention formats and measurement tools. Rather than suggesting the superiority of one approach over another, these comparisons highlight the need for future research to delineate the specific mechanisms of change within experiential therapies such as EFT and CFT in addressing self‐criticism.

### 3.4 Future Directions

Several limitations should be acknowledged. In addition to the limitations inherent in single‐group designs, the overall methodological quality of some included studies was suboptimal. The included studies were conducted across a limited range of regions, and participant samples were predominantly composed of women, raising questions about the generalisability of the findings. This reflects broader patterns of underrepresentation in psychotherapy research and highlights the need for more inclusive studies. Cultural context may influence how self‐criticism is understood and valued. In some settings, self‐criticism and shame may be viewed as socially normative or functional, whereas in others they may be regarded as maladaptive. Such differences may shape the meaning of self‐criticism and its responsiveness to EFT. Future research should therefore include more culturally diverse samples and examine how cultural beliefs moderate therapeutic outcomes.

## 4 Conclusion

In summary, this meta‐analysis provides preliminary evidence supporting the effectiveness of EFT in reducing self‐criticism, with observed effect sizes falling within the moderate‐to‐large range. These findings contribute promising empirical support to the transdiagnostic treatment literature and reinforce the clinical relevance of EFT in targeting self‐criticism as one of the core transdiagnostic factors potentially contributing to various mental health difficulties. However, further high‐quality, culturally sensitive and methodologically rigorous research is needed to replicate and extend these findings across more diverse populations.

## Funding

The authors have nothing to report.

## Ethics Statement

The authors have nothing to report.

## Conflicts of Interest

The authors declare no conflicts of interest.

## Supporting information


**Figure S1:** Effect Size Trend across Different r Values.
**Figure S2:** Leave‐One‐Out Sensitivity Analysis of Pre–Post EFT Effect Size for Self‐Criticism.
**Figure S3:** Leave‐One‐Out Sensitivity Analysis of RCT EFT Effect Size for Self‐Criticism.

**Table S1:** CPP70346‐sup‐0002‐supplementaryTable.docx. *Summary of Study Characteristics and Outcomes*.
**Table S2:** Risk of Bias Assessment Using ROB‐2 Tool.
**Table S3:** Quality Assessment of Included Single‐Group Pre‐Post Studies.
**Table S4:** Meta‐Analysis Results under Different Assumed Pre–Post Correlation Coefficients (r).

## Data Availability

This study used data extracted from previously published studies. No new datasets were generated. Analysis code is available from the authors upon reasonable request.
