# Supplementary material for: Effectiveness of Emotion‐Focused Therapy for Self‐Criticism: A Systematic Review and Meta‐Analysis
Source: Clin Psychol Psychother. 2026 Oct 4;33(5):e70346. doi: 10.1002/cpp.70346 (PMC13634925; doi:10.1002/cpp.70346)
Supplement: Supplementary file 1 [file CPP-33-e70346-s001.docx]

**Supplementary Table S1**

Summary of Study Characteristics and Outcomes

| Study (Author, Year) | Ethnicity | N | Pre_Mean | Pre_SD | Post_Mean | Post_SD | r (Pre-Post Correlation) | Dropout Rate (%) | Completion Rate (%) |
| --- | --- | --- | --- | --- | --- | --- | --- | --- | --- |
| Shahar et al. (2011) | Not reported (Israel/USA) | 10 | 28.40 | 5.10 | 18.20 | 7.55 | NR | 17% (2/12 dropped) | 83%（10/12） |
| Thompson & Girz (2019) | NR | 22 | 26.4 | 8.6 | 13.2 | 10.0 | NR | 12%（3/25） | 88%（22/25） |
| Stiegler et al. (2017) | Caucasian | 21 | 51.86 | 15.1 | 45.6 | 15.29 | NR | 12.5% (3/24) | 87.5% (21/24) |
| Shahar et al. (2017) | Not specified | 12 | 22.50 | 8.66 | 15.67 | 6.39 | NR | 8.3% (1/12) | 91.7% (11/12) |
| Baránková & Sládkovičová (2022) | Slovak/Czech | 32 | 34.78 | 4.75 | 29.03 | 5.74 | NR | 34.7%（34/98） | 65.3%（64/98） |
| Halamová et al. (2019) | Slovak | 19 | 19.00 | 5.95 | 15.21 | 5.77 | NR | 17%（4/23） | 83%（19/23） |
| Baránková & Vadurová (2022) | Slovak | 32 | 2.86 | 0.93 | 2.27 | 0.67 | NR | 0 | 100% |
| Baránková & Karpinský (2022) | Slovak | 37 | 23.67 | 12.09 | 21.43 | 11.73 | NR | 0 | 100% |
| López-Cavada et al. (2024) | Spanish | 21 | 22.4 | 7.4 | 16.1 | 7.4 | NR | 12.5% (3/24) | 87.5%（21/24） |
| Salasi et al., 2023 (EFT Group) | Iranian | 15 | 42.80 | 4.16 | 23.80 | 7.42 | NR | 0 | 100% |
| Halamová et al., 2022 (EFT-HP Group) | Slovak/Czech | 32 | 18.25 | 7.89 | 12 | 7.26 | NR | 74.8% (95/127) | 25.2% (32/127) |

Note. M = Mean; SD = Standard Deviation; r = Pre-post correlation; NR = Not reported. Dropout and completion rates are percentages calculated from original sample sizes (e.g., 17% = 2/12 dropped).

**Supplementary Table S2**

*Risk of Bias Assessment Using ROB-2 Tool*

| Studies | Baránková & Sládkovičová (2022) | Halamová et al. (2019) | Baránková & Vadurová (2022) | Baránková & Karpinský (2022) | López-Cavada et al. (2024) | Salasi et al., 2023 | Halamová et al., 2022 |
| --- | --- | --- | --- | --- | --- | --- | --- |
| 1.Randomization Process | Rating | Rating | Rating | Rating | Rating | Rating | Rating |
| Random sequence | PY | PY | PY | PY | Y | Y | PY |
| Allocation concealment | NI | NI | NI | NI | Y | Y | NI |
| Baseline imbalances | N | N | N | N | N | N | N |
| Domain 1 Judgment | Some Concerns | Some Concerns | Some Concerns | Some Concerns | Low | Low | Some Concerns |
| 2.Deviations from Interventions |  |  |  |  |  |  |  |
| Participant awareness | Y | Y | Y | Y | Y | PN | Y |
| Provider awareness | Y | Y | Y | Y | Y | Y | Y |
| Deviations due to trial context | PN | PN | PN | PN | PN | PN | PN |
| Impact of deviations | NA | NA | NA | NA | NA | NA | NA |
| Balance in deviations | NA | NA | NA | NA | NA | NA | NA |
| ITT analysis | PN | PN | Y | Y | Y | Y | Y |
| Impact of analysis failure | PY | PN | NA | NA | NA | NA | NA |
| Domain 2 Judgment | High | Some Concerns | Low | Low | Low | Low | Low |
| 3. Missing Outcome Data |  |  |  |  |  |  |  |
| High attrition | N | N | Y | Y | Y | Y | N |
| Missingness bias | PN | Y | NA | NA | NA | NA | PN |
| Missingness outcome-dependent | PY | N | NA | NA | NA | NA | NI |
| Likely missingness dependent | PY | NA | NA | NA | NA | NA | PN |
| Domain 3 Judgment | High | Low | Low | Low | Low | Low | Some concerns |
| 4.Outcome Measurement |  |  |  |  |  |  |  |
| Valid outcome tools | N | N | N | N | N | N | N |
| Consistent measurement | N | N | N | N | N | N | N |
| Assessor blinding | Y | Y | Y | Y | PY | NI | PY |
| Measurement bias | PY | PY | PY | PY | PY | PN | PY |
| Bias impact | NI | PN | PN | PN | PN | NA | PN |
| Domain 4 Judgment | High | Some concern | Some concern | Some concern | Some concern | Low | Some concern |
| 5. Reported Results |  |  |  |  |  |  |  |
| Pre-specified analysis | NI | NI | NI | NI | Y | PY | NI |
| Selective outcome reporting | N | N | N | N | N | N | N |
| Selective analysis reporting | N | N | N | N | N | N | N |
| Domain 5 Judgment | Some Concerns | Some Concerns | Some Concerns | Some Concerns | Low | Low | Some Concerns |
| Overall judgement | High | High | High | High | Some Concerns | Low | High |

*Note.* Y = Yes; PY = Probably Yes; PN = Probably No; N = No; NI = No Information; NA = Not Applicable.

| Criterion (Keyword) | Ben Shahar et al. (2011) | Thompson & Girz (2019) | Stiegler et al. (2017) | Ben Shahar et al. (2017) |
| --- | --- | --- | --- | --- |
| Study question clarity | Y | Y | Y | Y |
| Eligibility criteria description | Y | Y | Y | Y |
| Participant representativeness | N | Y | N | Y |
| Enrollment completeness | Y | Y | N | Y |
| Sample size adequacy | N | N | N | N |
| Intervention consistency | Y | Y | Y | Y |
| Outcome measure validity/reliability | Y | Y | Y | Y |
| Blinding of outcome assessors | N | N | N | N |
| Follow-up rate ≤ 20 % | Y | N | Y | Y |
| Statistical tests of pre–post changes | Y | Y | Y | Y |
| Multiple pre-/post measurements | N | Y | Y | Y |
| Group-level analysis adjustment | NA | Y | NA | NA |
| Quality Ratine | Fair | Fair | Fair | Good |

**Supplementary Table S3**

*Quality Assessment of Included Single-Group Pre-Post Studies*

*Note.* Y = Yes; N = No; NA = Not applicable; CD = Cannot determine; NR = Not reported.

**Supplementary Table S4**

*Meta-Analysis Results under Different Assumed Pre–Post Correlation Coefficients (r)*

| r Value | Hedges’ g | 95% CI | I² (%) | Tau² | p |
| --- | --- | --- | --- | --- | --- |
| 0.3 | -0.7320 | -0.9822, -0.4819 | 66.33 | 0.1112 | .0009 |
| 0.5 | -0.8638 | -1.1551, -0.5725 | 73.39 | 0.1662 | .0001 |
| 0.7 | -1.0999 | -1.4615, -0.7383 | 80.40 | 0.2796 | < .0001 |

*Note.* Pooled effect sizes (Hedges’ g) were computed using a random-effects model. Heterogeneity statistics (*I*² and Tau²) indicate increasing variability with larger *r* assumptions.
