# Supplementary material for: Effectiveness of Emotion‐Focused Therapy for Self‐Criticism: A Systematic Review and Meta‐Analysis
Source: Clin Psychol Psychother. 2026 Oct 4;33(5):e70346. doi: 10.1002/cpp.70346 (PMC13634925; doi:10.1002/cpp.70346)
Supplement: Supplementary file 2 [file CPP-33-e70346-s002.docx]

For each specific study, the variation in effect size under different assumed values of r is shown in Figure 3. Among these, the studies by Shahar et al. (2011), Thompson et al. (2019), and Salasi et al. (2023) were sensitive to the value of *r*, meaning that their effect sizes were more affected by changes in *r*. They were strongly influenced by the assumed *r* values, showing relatively greater changes as *r* varied. For example, Shahar et al.’s (2011) research showed a change in effect size from -1.5 to -2.8 when the assumed value of *r* increased from 0.3 to 0.7. In addition, Hedges’ g decreased as *r* increased, exhibiting a clear slope and relatively wide confidence intervals.

Halamová et al. (2019), Baránková and Vadurová (2022), and Lopez-Cavada et al. (2024) showed effect sizes that were moderately influenced by the assumed *r*, with the range of variation in effect size being around -0.5. The remaining five studies’ effect size remains stable, indicating that their effect size Hedges’ *g* was slightly influenced by *r*. Some studies, such as Baránková and Karpinský (2022), showed almost do not fluctuation with changes of *r*, demonstrating their robustness and internal consistency.

All in all, Stiegler et al. (2017), Shahar et al. (2017), the two studies by Baránková et al. (2022), and Halamová et al. (2022) showed that their effect sizes were less impacted by the assumed *r* and remained stable with changes in *r*, demonstrating their robustness.

**Supplementary Figure S1** *Effect Size Trend across Different r Values*

**Supplementary Figure S2** *Leave-One-Out Sensitivity Analysis of Pre–Post EFT Effect Size for Self-Criticism*

**Supplementary Figure S3** *Leave-One-Out Sensitivity Analysis of RCT EFT Effect Size for Self-Criticism*
